# Care of the Eyes

**Published:** 1857-12

**Authors:** 


### CARE OF THE EYES.

CRAWFORD, the celebrated sculptor, had an inveterate habit of reading in a reclining position: one eye has been taken out in consequence of a cancerous tumor forming behind it, and his life has paid the forfeit, after years of suffering, and the expenditure of a large amount of money.

PRESCOTT, the Historian, in consequence of a disorder of a nerve, by which the eyes were rendered useless for all writing purposes, could not use a pen, as he was unable to see when it failed to make a mark, for want of ink; nor could he distinguish the lines or edges of his paper; yet, with these disadvantages, he wrote all his *historicals*, using an agate stylus on carbonated paper, being guided as to the lines or edges by brass wires drawn through a wooden frame: but, with all these hindrances, he has made himself one of the most readable of modern historians, and earned a fortune besides.

To avoid these and similar calamities, we urge upon the young, especially, never to use the eyes by any artificial light, where nicety of sight is required, nor to use them in any strained position, or while riding in rail-cars or carriages.

We urge upon all parents, in view of the many incurable eye diseases, to caution their children against reading by twilight, that is, not before sunrise nor after sunset. It would be greatly better not to allow them to read or sew by any artificial light; but if that is unavoidable, let it be imperative that they cease by nine o'clock at night in summer, and by ten at farthest, in the winter. It is a most inexcusable folly, and will, sooner or later, bring its punishment, to read or sew by gas, or lamp, or candle light, and then sleep after daylight next morning, as a habit. To persons of all ages it is a most injurious practice.

